# Supplementary material for: Distribution of Bacterial Endosymbionts of the Cardinium Clade in Plant-Parasitic Nematodes
Source: Int J Mol Sci. 2023 Feb 2;24(3):2905. doi: 10.3390/ijms24032905 (PMC9918034; doi:10.3390/ijms24032905)
Supplement: Supplementary file 1 [file ijms-24-02905-s001.zip › Supplementary materials 1.pdf]

International Journal of
Molecular Sciences

MDPI

Supplementary materials

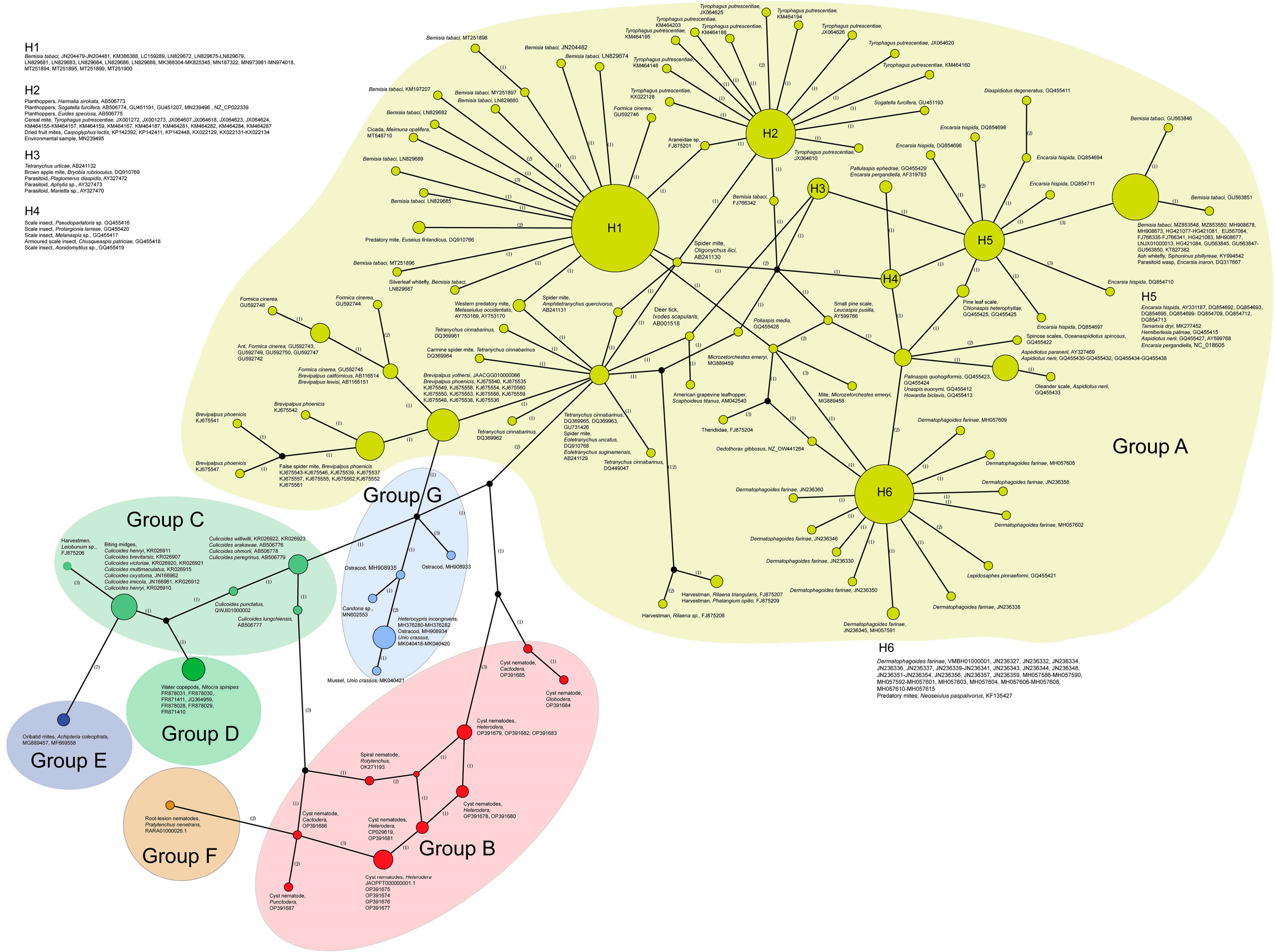

Figure S1. Statistical parsimony network showing the 16S rRNA gene-based phylogenetic relationships between representatives of *Cardinium* clade from different host organisms (432 sequences, alignment length - 461 bp). Different colours mark the sequences of each group. Pies (circles) represent sequences with the same haplotype and their size is proportional to the number of these sequences. Numbers of nucleotide differences between the sequences are indicated on lines connecting the pies. Small black circles represent missing haplotypes.

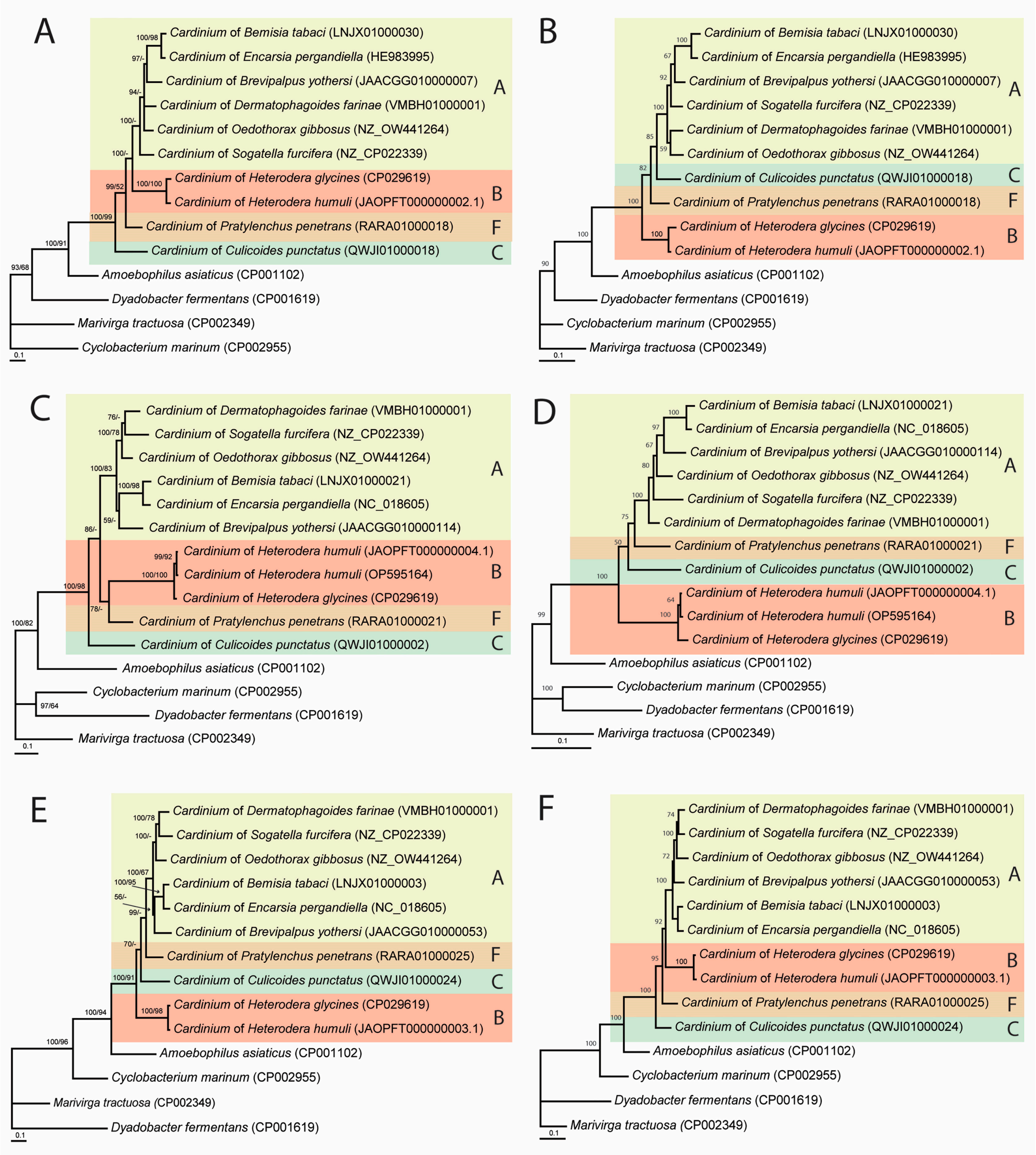

Figure S2. Phylogenetic relationships among representatives of *Cardinium* clade. Bayesian 50% majority rule consensus trees as inferred from alignments. A: *sufB* nucleotide (alignment length - 1397 bp) (A) and amino acid (alignment length - 465 aa) (B) sequence alignments; B: *groEL* nucleotide (1644 bp) (C) and amino acid (533 aa) (D) sequence alignments; C: *fusA* nucleotide (2113 bp) (E) and amino acid (701 aa) (F) sequence alignments. Posterior probabilities (BI) and bootstrap values (ML) more than 50% are shown at branching points.

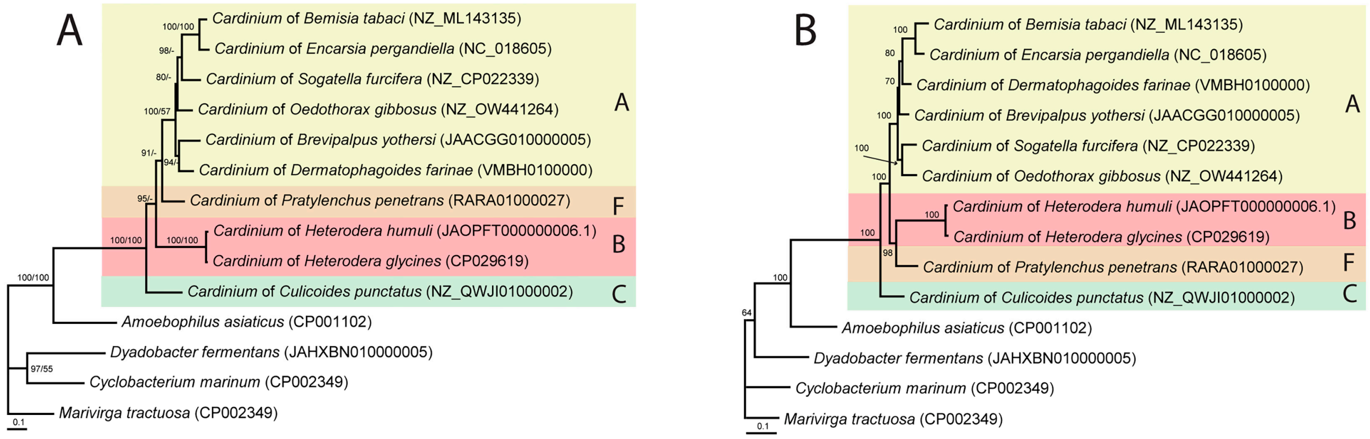

Figure S3. Phylogenetic relationships among representatives of *Cardinium* clade. Bayesian 50% majority rule consensus trees as inferred from *infB* nucleotide (alignment length - 1499 bp) (A) and amino acid (alignment length - 499 aa) (B) sequence alignments. Posterior probabilities (BI) and bootstrap values (ML) more than 50% are shown at branching points.

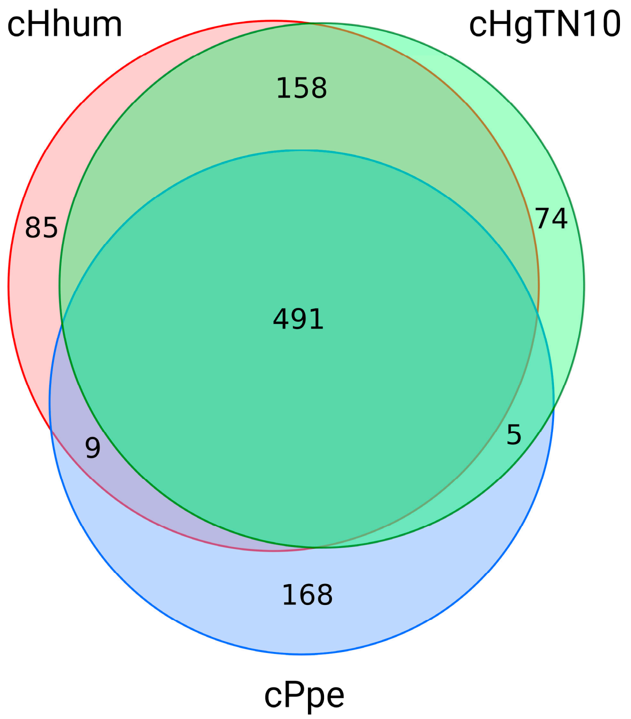

Figure S4. Genome content comparisons among three strains of the *Cardinium* clade. Numbers inside each subspace represent the number of orthologous gene clusters assigned to the subspace. cHhum - *Cardinium* of *Heterodera humuli*; cHgTN10 - *Cardinium* of *Heterodera glycines*; cPpe - *Cardinium* of *Pratylenchus penetrans* (gene list is in the Supplementary Table S3).

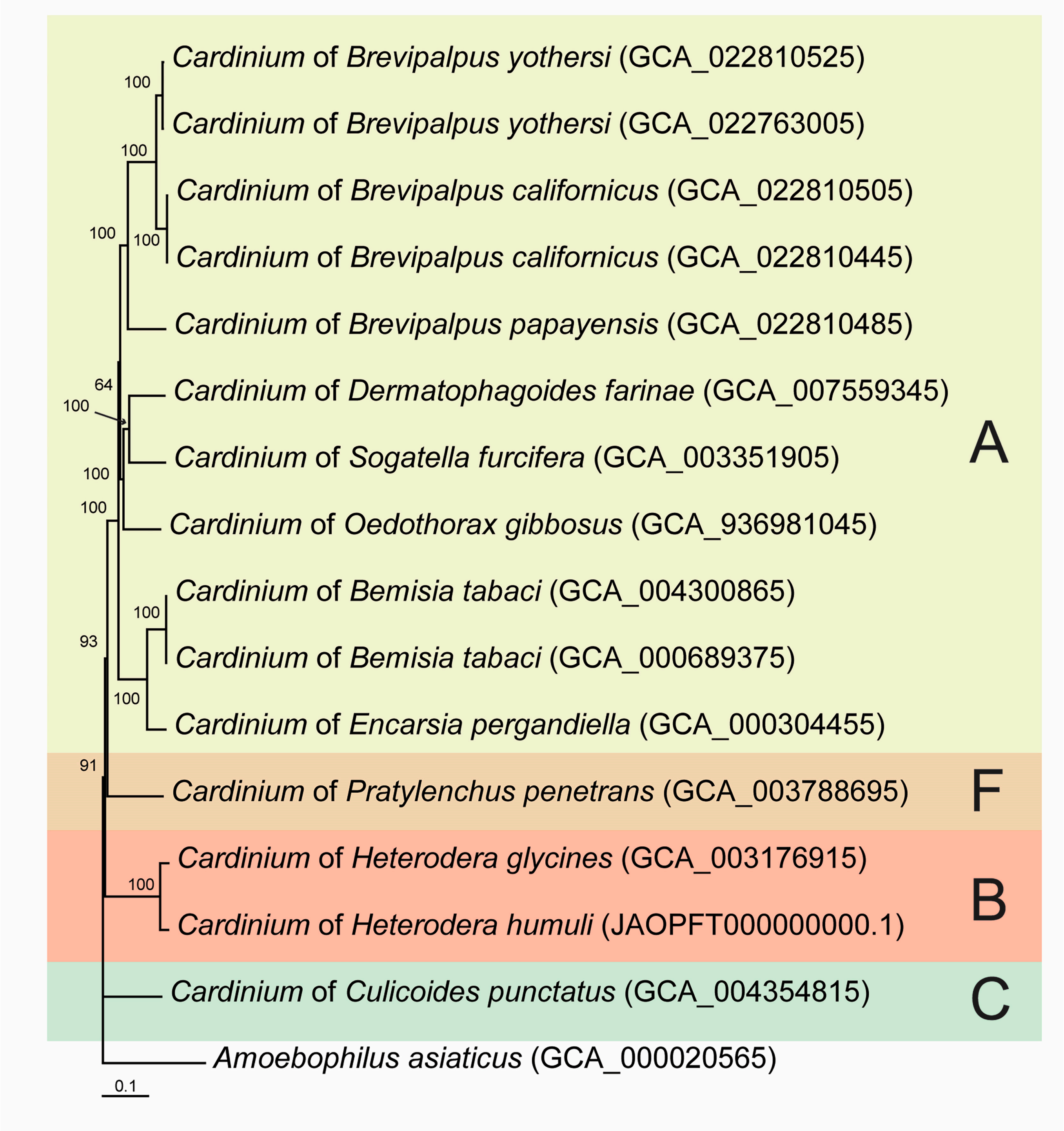

Figure S5. Phylogenomic tree based on 15 bacterial genomes of the *Cardinium* clade. The tree is drawn to scale, with branch lengths measured in the estimated number of substitutions per site. Branch support values (rate of elementary quartets) above 50 are indicated at the branch points. The genomic sequence of "*Candidatus* Amoebophilus asiaticus" 5a2 (CP001102.1 = GCA_000020565) served as an outgroup.

Table S1. PCR screening of nematode species and populations for endosymbionts of *Cardinium* and relatives.

Species	Sample code	Location	PCR with specific primers
**Order Tylenchida, Family Heteroderidae, Subfamily Heteroderinae**			
Heterodera arenaria	CD1440	Italy	-
Heterodera australis	CD1284	Australia, Victoria	-
Heterodera avenae	CD1488	Turkey	-
Heterodera avenae	CD1456	Tunisia, Siliana	+
Heterodera avenae	CD1504	Tunisia, Siliana	-
Heterodera avenae	CD1982	Egypt, Al Tasa	-
Heterodera carotae	CD2402	Mexico	-
Heterodera cruciferae	CD2865	USA, California, Yuba County	-
Heterodera fici	CD2067	USA, California, Fresno County	-
Heterodera filipjevi	CD1477	Ukraine, Chabany	-
Heterodera filipjevi	CD1617	Syria, El-Hassake, Khanka	-
Heterodera filipjevi	CD2364	Iran, Khuzestan, Ramshir	-
Heterodera filipjevi	CD1489	Germany, Flensburg	-
Heterodera glycines	CD1430	USA, North Carolina	-
Heterodera goettingiana	CD3123a	France, Antibes	+
Heterodera guangdongensis	CD2237	Viet Nam	-
Heterodera hordecalis	CD1486	Sweden	-
Heterodera humuli	CD3144b-2	Russia, Moscow	+
Heterodera latipons	CD2047	Turkey, Kilis, Merkez	+
Heterodera latipons	CD1992	Turkey, Elbistan	+
Heterodera latipons	CD2366	Iran, Zanjan	-
Heterodera mani	CD1237	USA, California	-
Heterodera mani	CD956	USA, Washington	-
Heterodera mediterranea	CD3246	Spain, Saler, Valencia	+
Heterodera mediterranea	CD3118a	Spain, Vejer, Cádiz	+
Heterodera mediterranea	CD3243	Spain, Puebla de Cazalla, Sevilla	-
Heterodera mediterranea	CD3242	Spain, Sanlúcar de Barrameda, Cadiz	-
Heterodera mediterranea	CD3251	Spain, Hinojos, Huelva	-
Heterodera mediterranea	CD3252	Spain, Villaviciosa, Cordoba	-
Heterodera mediterranea	CD3253	Spain, Hinojos, Huelva	-
Heterodera mediterranea	CD3254	Spain, Tarifa, Cadiz	-
Heterodera mediterranea	CD3255	Spain, Puente Genil, Cordoba	-
Heterodera pratensis	CD2398	Germany	-
Heterodera ripae	CD3144b-4	Russia, Moscow	+
Heterodera sacchari	CD2960	Ghana, Ashani region	-
Heterodera salixophila	CD3259	Belgium, Nieuwpoort	-
Heterodera salixophila	CD3258	Ukraine, Kherson	+
Heterodera schachtii	CD436	USA, California	-
Heterodera schachtii	CD1436	USA, California, Santa Barbara	-
Heterodera schachtii	CD1530	Ukraine, Vinnitsa region	-
Heterodera sturhani	CD2360a	China	+
Heterodera trifolii	CD1083	USA, California	-
Heterodera trifolii	CD1438	Russia, Nizhny Novgorod region	-
Heterodera sp.1	CD3011	Mexico, Hidalgo, Singuilucan	-

Heterodera sp.2	CD3117	Belgium	-
**Subfamily Punctoderinae**			
Cactodera rosae	CD329	Mexico	+
Cactodera torreyanae	CD1057	Mexico	-
Cactodera sp.	CD3612	Mexico	+
Globodera artemisiae	CD2193	Germany	-
Globodera artemisiae	CD2178	Russia, Primorsky Krai	-
Globodera capensis	CD1892	South Africa	-
Globodera ellingtonae	CD1988	USA, Oregon	-
Globodera mexicana	CD2809	Mexico	-
Globodera mexicana	CD2784	Mexico, State of Mexico,	-
Globodera pallida	CD2740	Netherlands	-
Globodera pallida	CD2741	France	-
Globodera pallida	CD2577	Spain, Tenerife	-
Globodera pallida	CD2595	Bolivia, La Paz, Los Andes	-
Globodera pallida	CD2744	USA, Idaho	-
Globodera pallida	CD2547	Costa Rica	-
Globodera rostochiensis	CD2568b	Bolivia, Cochabamba, Saavedra	+
Globodera rostochiensis	CD2167	Russia, Smolensk	
Globodera rostochiensis	CD2618	Bolivia, Potosi, Laguna Pampa	-
Globodera rostochiensis	CD2682	Russia, Belgorod region	-
Globodera rostochiensis	CD2561	Bolivia, Las Paz, Los Andes, Seviruyo	-
Globodera rostochiensis	CD2200	Germany, Harmerz	-
Globodera rostochiensis	CD2165	Kyrgyzstan	-
Globodera rostochiensis	CD2840	South Africa, Gauteng	-
Globodera rostochiensis	CD2619	Bolivia, Cochabamba, Tapacari	-
Globodera rostochiensis	CD2593	Bolivia, Cochabamba, Arani	-
Globodera tabacum	CD2692	USA	-
Globodera tabacum	CD2206	USA, Virginia	-
Globodera zelandica	CD2194	New Zealand	-
Globodera zelandica	CD2184	New Zealand	-
Punctodera chalcoensis	CD2813	Mexico	+
Punctodera punctata	CD2715	USA, Michigan	-
Punctodera stonei	CD3581	USA, Virginia	-
Punctodera stonei	CD955	USA, Washington	-
Punctodera sp.	CD3549	USA, California, Santa Clara, San Jose	-
**Subfamily Ataloderinae**			
Atalodera sp.	CD1355	USA, California	-
**Subfamily Meloidoderinae**			
Cryphodera sinensis	CD2230	Viet Nam	-
Meloidodera floridensis	CD1957	USA, Florida	-
**Subfamily Verutinae**			
Verutus volvingentis	CD2523	USA, Florida	-
**Family Hoplolaimidae**			
Helicotylenchus digonicus	CD1072	USA, Kansas	-
Helicotylenchus dihystera	CD508	USA, Florida, Goulds	-
Helicotylenchus dihystera	CD600	USA, Florida, Homestead	-
Helicotylenchus microlobus	CD1327	USA, Arizona	-
Helicotylenchus paxilli	CD1227	USA, California	-

Helicotylenchus pseudorobustus	CD599	USA, California, Fresno	-
Helicotylenchus pseudorobustus	CD1234	USA, California	-
Helicotylenchus sp.	CD1052	USA, California	-
Hoplolaimus galeatus	CD3357	USA, Maryland	-
Hoplolaimus galeatus	CD3630	USA, Florida	-
Aorolaimus christiei	CD1495	USA, Florida	-
Rotylenchus brevicaudatus	CD425	Australia, Brisbane	-
Rotylenchus buxophilus	CD827	USA, California, Napa County,	-
Rotylenchus robustus	CD858	USA, California, Marin County	-
Rotylenchus robustus	CD880	USA, California, Tomales	-
Rotylenchus pumilus	CD903	USA, California, Santa Clara County	-
Scutellonema brachyurus	CD1804	USA, California, Riverside	-
Scutellonema cavenessi	CD1470	USA, Florida, Homestead	-
**Family Rotylenchulidae**			
Rotylenchulus reniformis	CD746	USA	
Rotylenchulus parvus	CD317	South Africa	-
**Family Pratylenchidae**			
Nacobbus aberrans	CD413	Mexico, Saltillo	-
Pratylenchus bolivianus	CD1032	Costa Rica	-
Pratylenchus coffeae	CD3703	Puerto Rico	-
Pratylenchus coffeae	CD3575	Puerto Rico	-
Pratylenchus convallariae	CD1813	USA	-
Pratylenchus hippeastri	CD3356	USA, Florida	-
Pratylenchus neglectus	CD1735	Bolivia	-
Pratylenchus penetrans	CD2859	USA, California	-
Pratylenchus pinguicaudatus	CD2361	UK	-
Pratylenchus zeae	CD531	USA, Florida, Milton	-
Pratylenchus sp.1	CD871	USA, Kansas, Manhattan	-
Pratylenchus sp.2	CD3135	USA, Oregon	-
Radopholus similis	CD3662	USA, Florida	-
**Family Meloidogynidae**			
Meloidogyne enterolobii	CD3705	Puerto Rico	-
Meloidogyne hispanica	CD2439	USA, Florida, Palm Beach County	-
Meloidogyne incognita	CD3545	USA, California, Merced	-
Meloidogyne naasi	CD2158	USA, California	-
**Family Hemicycliophoridae**			
Hemicycliophora poranga	CD1835	USA, California, Marin County	-
Hemicycliophora robbinsi	CD656	USA, Florida, Fort Lauderdale	-
Hemicycliophora sp.	CD2274	Canada	-
**Family Paratylenchidae**			
Paratylenchus hamatus	CD315	USA, California, Kern County, Wasco	-
Paratylenchus straeleni	CD3708	USA, Florida	-
Paratylenchus straeleni	CD899	USA, California, Napa County	-
**Family Tylenchulidae**			
Trophotylenchus arenarium	CD3472	USA, California	-
**Family Criconematidae**			
Hemicriconemoides californianus	CD1054	USA, California, Berkley	-
Hemicriconemoides cocophilus	CD1496	USA, Florida, Key West	-
Hemicriconemoides	CD1513	USA, Florida, Miami, Black Point	-

strictathecatus			
Mesocriconema ornatum	CD2383	USA, Florida	-
Mesocriconema xenoplax	CD2024	USA, Florida	-
**Family Dolichodoridae**			
Dolichodorus sp.	CD3713a	USA, Florida	-
Dolichodorus sp.	CD3746	USA, Florida	-
**Family Telotylenchidae**			
Paratrophurus bursifer	CD2257	Belgium	-
Paratrophurus loofi	CD362	Spain, Jaén, Coto Rios	-
Tylenchorhynchus agri	CD712	USA, Florida, Delray Beach	-
Tylenchorhynchus clarus	CD197	USA, California, Colusa County	-
**Order Aphelenchida, Family Aphelenchidae**			
Bursaphelenchus juglandis	CD2937	USA, California, Fresno County	-
Bursaphelenchus mucronatus	CD1421	Russia	-
Bursaphelenchus mucronatus	CD1409	Russia	-

+ - amplicon present; - no amplification.

Table S2. Number of reads related to *Cardinium* in the studied cyst nematode samples.

Characteristics	CD3144_Hum	CD3144_Rip	CD3144
No. of all reads	143,341,468	141,231,712	139,187,652
No. of read for *Cardinium*	3,523,302	130,016	163,292
Percentage of read for *Cardinium*	2.46	0.09	0.12
Genome coverage (×)	452	17	22

ANI between assemblies of *Cardinium* of *Heterodera humuli* is more than 99.4%.

Table S3. List of orthogroups for three *Cardinium* strains of plant-parasitic nematodes (orthogroups.xlsx file).

Table S4. ANI and dDDH values for *Cardinium* cHhum strain from the hop cyst nematode, *Heterodera humuli,* and related bacteria.

Assembly Accession Number	GenBank Accession Number	Strain	ANI	dDDH
GCA_000304455.1	HE983995.1, HE983996.1	Cardinium endosymbiont cEper1 of *Encarsia pergandiella*	70.12	22.5
GCA_000689375.1	CBQZ000000000.1	Cardinium endosymbiont cBtQ1 of *Bemisia tabaci*	70.385	20.1
GCA_003176915.1	CP029619.1	Candidatus Cardinium hertigii cHgTN10	93.96	55
GCA_003351905.1	CP022339.1	Cardinium endosymbiont of *Sogatella furcifera* cSfur	70.225	20.6
GCA_003788695.1	RARA00000000.1	Candidatus Cardinium hertigii Pp_1	70.72	19
GCA_004300865.1	LNJX00000000.1	Cardinium endosymbiont of *Bemisia tabaci*	70.245	20
GCA_004354815.1	QWJI00000000.1	Cardinium endosymbiont of	68.895	20.4

		Culicoides punctatus cCpun		
GCA_007559345.1	VMBH00000000.1	Cardinium endosymbiont of *Dermatophagoides farinae*	70.115	19.9
GCA_022763005.1	JAALXE000000000.1	Candidatus Cardinium hertigii cByotB1	69.93	20
GCA_022810445.1	JAACGD000000000.1	Candidatus Cardinium sp. cBcalN2	70.025	21.1
GCA_022810485.1	JAACGF000000000.1	Candidatus Cardinium sp. cBpapB1	70.225	21.6
GCA_022810505.1	JAACGE000000000.1	Candidatus Cardinium sp. cBcalN1	70.04	21.2
GCA_022810525.1	JAACGG000000000.1	Candidatus Cardinium sp. cByotN1	70.19	20.7
GCA_936981045.1	OW441264.1	Cardinium endosymbiont of *Oedothorax gibbosus*	70.695	22.4
